# Role of GalNAc4S-6ST in Astrocytic Tumor Progression

**DOI:** 10.1371/journal.pone.0054278

**Published:** 2013-01-17

**Authors:** Tatsuya Kobayashi, Huimin Yan, Yasuhiro Kurahashi, Yuki Ito, Hiroshi Maeda, Tsuyoshi Tada, Kazuhiro Hongo, Jun Nakayama

**Affiliations:** 1 Department of Molecular Pathology, Shinshu University Graduate School of Medicine, Matsumoto, Japan; 2 Department of Neurosurgery, Shinshu University School of Medicine, Matsumoto, Japan; 3 Department of Medical Education, Shinshu University School of Medicine, Matsumoto, Japan; 4 Central Research Laboratories, Seikagaku Corporation, Higashiyamato, Japan; Florida International University, United States of America

## Abstract

*N*-Acetylgalactosamine 4-sulfate 6-*O*-sulfotransferase (GalNAc4S-6ST) is the sulfotransferase responsible for biosynthesis of highly sulfated chondroitin sulfate CS-E. Although involvements of CS-E in neuronal cell functions have been extensively analyzed, the role of GalNAc4S-6ST in astrocytic tumor progression remains unknown. Here, we reveal that GalNAc4S-6ST transcripts were detected in astrocytic tumors derived from all 30 patients examined using quantitative reverse transcription-PCR analysis. Patients with high GalNAc4S-6ST mRNA expression had significantly worse outcome compared with patients with low expression, and multivariate survival analysis disclosed that GalNAc4S-6ST is an independent poor prognostic factor for astrocytic tumors. We then tested whether CS-E enhanced haptotaxic migration of glioblastoma U251-MG cells that endogenously express both the CS-E’s scaffold tyrosine phosphatase ζ (PTPζ) and GalNAc4S-6ST, in the presence of CS-E’s preferred ligands, pleiotrophin (PTN) or midkine (MK), using a modified Boyden chamber method. Haptotaxic stimulation of cell migration by PTN was most robust on control siRNA-transfected U251-MG cells, while that enhancing effect was cancelled following transduction of GalNAc4S-6ST siRNA. Similar results were obtained using MK, suggesting that both PTN and MK enhance migration of U251-MG cells by binding to CS-E. We also found that PTPζ as well as PTN and MK were frequently expressed in astrocytic tumor cells. Thus, our findings indicate that GalNAc4S-6ST mRNA expressed by astrocytic tumor cells is associated with poor patient prognosis likely by enhancing CS-E-mediated tumor cell motility in the presence of PTN and/or MK.

## Introduction

Chondroitin sulfate (CS) is a linear glycan chain composed of repeating disaccharide units of glucuronic acid (GlcA) and *N*-acetylgalactosamine (GalNAc) and attached to specific scaffolds as glycosaminoglycans, thus forming a major extracellular matrix proteoglycan in the brain [1]. CS disaccharide units are variably sulfated by sulfotransferases at specific GlcA and/or GalNAc positions classified as 5 motifs, A-, B-, C-, D-, and E-units based on the position of sulfation [2]. Among them, the highly sulfated E-unit (CS-E), composed of repeating GlcAβ1-3GalNAc(4,6-bis-SO_4_) disaccharide units with a β1,4-linkage, is unique, because its negative charge functions in several biological processes by preferential binding to heparin-binding growth factors such as midkine (MK) and pleiotrophin (PTN) [3], [4], basic fibroblast growth factors (bFGF) [3], E/P-selectin, and chemokines [5].

Roles of CS-E in neuronal cell functions have been extensively analyzed. Sato et al (2008) demonstrated that CS-E prevents excitatory amino acid-induced neuronal cell death [6]. The role of CS-E in its ability to enhance or impede neurite outgrowth is still controversial; some studies reported CS-E as being growth promoting [7], [8], while recent studies using a homogeneous population of CS-E [9] or using immobilized CS-E spots [10] showed that CS-E is a potent inhibitor of neurite outgrowth. In tumors, CS-E inhibits P-selectin binding to the metastatic breast cancer cell line 4T1 *in vitro* in a Ca^2+^-independent and sialyl Lewis X/A-independent manner [11]. Since P-selectin is found within the Weibel-Palade bodies of endothelial cells and α-granules of platelets [12], CS-E on tumor cells may facilitate metastasis by binding to P-selectin present in endothelial cells and/or platelets. In addition, ten Dam et al (2007) demonstrated that CS-E is strongly expressed in human ovarian adenocarcinomas, while it is not expressed in normal ovary by generating antibody against CS-E [13]. More recently, the same group also showed that CS-E functions in metastasis of a Lewis lung carcinoma cell line [14], and that CS-E expressed in murine osteosarcoma LM8G7 cells is involved in focal formation of liver tumors [15].

*N*-Acetylgalactosamine 4-sulfate 6-*O*-sulfotransferase (GalNAc4S-6ST) is a specific sulfotransferase responsible for biosynthesis of CS-E from chondroitin sulfate A (CS-A) through transfer of a sulfate group to position C6 of GalNAc(4SO_4_) in CS-A [16], [17]. Recently, Ohtake-Niimi et al. (2010) developed GalNAc4S-6ST-deficient mice and found that GalNAc4S-6ST is the sole sulfotransferase that forms CS-E, and that the level of mast cell proteases is decreased in bone marrow-derived mast cells of knockout mice [18].

Although GalNAc4S-6ST is widely expressed in the developing brain [1], it has not been known whether GalNAc4S-6ST is expressed in brain tumors. In the present study, we use quantitative reverse transcription-PCR (RT-PCR) to show that GalNAc4S-6ST mRNA is detected in astrocytic tumor and that its expression level is significantly associated with poor patient prognosis likely by enhancing CS-E-mediated tumor cell motility in the presence of PTN and/or MK.

## Materials and Methods

### Ethics Statement

The experimental protocol and use of all human pathology specimens for research were approved by the Ethical Committee of Shinshu University School of Medicine (Matsumoto, Japan). The Ethical Committee also granted a waiver of informed consent to use the formalin-fixed and paraffin-embedded tissue specimens retrieved from the pathology file of the Shinshu University Hospital, because the diagnostic use of the samples was completed before the study, and thus no risk to the involved patients was predicted. In addition, these samples were coded to protect patient anonymity. For the use of fresh tumor samples, written informed consent was obtained from all the participants before study, and these samples were coded to protect patient anonymity.

### Patients

Astrocytic tumors surgically resected from 34 patients were analyzed for the present study. Among them, tissue specimens from 30 patients whose prognoses had been followed for at least for 5 years after surgical resection of the tumor at the Shinshu University Hospital, were retrieved from the pathology file of the same hospital. According to the WHO classification [19], samples included pilocytic astrocytoma (astrocytoma grade I) (2 cases), diffuse astrocytoma (astrocytoma grade II) (5 cases), anaplastic astrocytoma (astrocytoma grade III) (8 cases), and glioblastoma (astrocytoma grade IV) (15 cases). For each patient, a representative portion of the tumor was examined. All tissue samples had been fixed in 20% formalin buffered with 0.1 M phosphate buffer (pH 7.4) at room temperature for 48 hours and embedded in paraffin. For biochemical analysis, fresh tumor samples were obtained from other 4 patients (3 cases of diffuse astrocytoma and 1 of glioblastoma) and stored at −80°C until use.

### Cell Line

Human glioblastoma U251-MG cells were kindly provided by Professor Jun Yoshida, Department of Neurosurgery, Nagoya University Graduate School of Medicine, Nagoya, Japan [20]. Cells were cultured in Dulbecco’s modified Eagle medium (DMEM; Life Technologies, Carlsbad, CA, USA) supplemented with 10% heat-inactivated fetal bovine serum (FBS; HyClone Laboratories, Logan, UT, USA) at 37°C in 5% CO_2_. Preliminary experiment using RT-PCR carried out prior to this study indicated that U251-MG cells endogenously express GalNAc4S-6ST mRNA (personal communications TK and JN).

### RNA Isolation and Quantitative RT-PCR

Total RNA was extracted from tissue embedded in paraffin blocks as described [21]. Briefly, 6 slices of 5 µm-thick tumor sections were transferred to a 1.5 ml-tube, and then 1 ml of Hemo De (FALMA, Tokyo, Japan) was added to each tube, which was then agitated on a wave shaker at room temperature for 20 minutes and centrifuged at 20,000×*g* for 20 minutes. The supernatant was immediately removed, and the step repeated 3 times. Next, 1 ml of 100% ethanol was added to the same tube and agitated. All tubes were shaken again at room temperature for 15 minutes, centrifuged at 20,000×*g* for 20 minutes, and immediately the supernatant was discarded, a step repeated three times. The pellet was dried and dissolved in 50 µl of solution containing 1 µg/µl proteinase K (Nacalai tesque, Kyoto, Japan), 20mM Tris-HCl (pH8.0), 0.15M NaCl, 5 mM EDTA, and 1% sodium dodecyl sulfate. Tubes were capped and incubated at 37°C overnight.

Total RNA was isolated from astrocytic tumors thus prepared as well as 2.5×10^6^ glioblastoma U251-MG cells as control using an RNeasy Mini Kit (Qiagen, Hilden, Germany), according to the manufacturer’s instructions. To remove genomic DNA, 15 µl of purified total RNA was digested with 2.5 µl of 10 U/µl RNase-free DNase I (Roche, Penzberg, Germany) at 37°C for 2 hours and heated at 70°C to inactivate the enzyme. Samples were then denatured at 70°C for 10 minutes and hybridized with 1 µl of 0.5 µg/µl random primers (Promega, Madison, WI, USA). For single-strand cDNA synthesis, total RNA was reverse transcribed using 1 µl of 200 U/µl SuperScript III (Invitrogen, Carlsbad, CA, USA), 2.5 µl of a 2.5 mM dNTP mixture, 0.5 µl of 0.1 M dithiothreitol, and 1 µl of 40 U/µl RNasin Plus RNase inhibitor (Promega) at 50°C for 1 hour and then heated to 70°C for 10 minutes. First-strand cDNA served as template for quantitative RT-PCR.

Quantitative RT-PCR analysis of GalNAc4S-6ST mRNA was achieved using the 7300 Real time PCR system (Applied Biosystems, Foster City, CA, USA), as described with a minor modification [21]. Briefly, a 50 µl solution containing 25 µl of 2× TaqMan universal master mix (Applied Biosystems), 0.5 µl of cDNA, and 2.5 µl of premixed reagents containing primers and a TaqMan probe for GalNAc4S-6ST (Hs00248144_m1; Applied Biosystems) or β_2_-microglobulin (B2M) (Hs99999907_m1; Applied Biosystems) were added to each well of 96-well optical plates. Plates were heated to 50°C for 2 minutes and 95°C for 10 minutes and then subjected to 55 thermal cycles (95°C for 15 seconds, 60°C for 1 minute). Absence of genomic DNA contamination was confirmed by amplifying samples without reverse transcriptase. Relative expression of GalNAc4S-6ST mRNA was normalized to B2M mRNA, and a comparative CT value was determined by defining the expression level of GalNAc4S-6ST mRNA in glioblastoma U251-MG cells as 1.0. Assays were performed in duplicate.

### *In Situ* Hybridization

Four tumor specimens with varying degrees of GalNAc4S-6ST mRNA expression as determined by quantitative RT-PCR were selected and analyzed by *in situ* hybridization, as described [21]. Briefly, digoxigenin (DIG)-labeled antisense and sense RNA probes were prepared from a plasmid containing a segment of human GalNAc4S-6ST (nucleotides 801–950; the first nucleotide of the initiation codon is defined as 1) by *in vitro* transcription. Then, 7 µm-thick tissue sections were deparaffinized in Hemo De (FALMA), hydrated using an ethanol/water series, neutralized with 0.2 M HCl for 20 minutes, treated with 50 µg/ml proteinase K (Amresco, Solon, OH, USA) at 37°C for 30 minutes, and postfixed with 4% paraformaldehyde. Tissue slides were rinsed in 0.2% glycine for quenching and acetylated with 0.25% acetic anhydride in 0.1 M triethanolamine (pH 8.0). Hydrated slides were defatted with chloroform and air-dried. Sections were prehybridized with 50% deionized formamide/2× SSC at 45°C for 1 hour and then hybridized with 1 µg/ml of GalNAc4S-6ST antisense or sense probe in 50% deionized formamide, 2.5 mM EDTA (pH8.0), 0.3 M NaCl, 1× Denhardt’s solution, 10% dextran sulfate, and 1 mg/ml brewer’s yeast tRNA at 45°C for 36 hours.

After hybridization, slides were washed in 50% formamide/2× SSC for 1 hour at 45°C and digested with 10 mg/ml RNase A (Amresco) in RNase buffer (0.5 M NaCl, 10 mM Tris-HCl) at 37°C for 30 minutes, followed by washing in 50% formamide/2× SSC and then 50% formamide/1× SSC at 37°C for 1 hour. After washing, sections were subjected to immunohistochemistry to detect hybridized probes with an alkaline phosphatase-conjugated anti-digoxigenin (DIG) antibody (Roche). The alkaline phosphatase reaction was carried out with 5-bromo-4-chloro-3-indolyl phosphate and nitroblue tetrazolium in the presence of 10% polyvinyl alcohol at 4°C overnight.

### Biochemical Analysis

To detect CS-E in astrocytoma, fresh brain tumor samples (22 to 122 mg wet weight) removed from 4 patients were subjected to biochemical analysis. After lyophilization, samples were digested with 150 µl of 2.5% actinase E (Kaken Pharmaceutical Co., Tokyo, Japan) at 55°C for 24 hours, boiled at 100°C for 5 minutes, and then centrifuged at 3,000 rpm for 10 minutes. Fifty µl of solution containing 250 mU of chondroitinase ABC (Seikagaku Co., Tokyo, Japan) and 25 mU of chondroitinase AC-II (Seikagaku Co.), 80 µl of 100 mM Tris-HCl buffer (pH 8.0), and 170 µl of distilled water were added to 100 µl of tissue sample supernatant and incubated at 37°C for 2 hours. Each reaction solution was ultrafiltered using a Nanosep with a molecular weight cutoff of 10,000 (Pall, Port Washington, NY, USA), and then filtrates were analyzed by high performance liquid chromatography (HPLC) according to the method of Shinmei et al [22]. The HPLC Model 2000 (JASCO, Easton, MD, USA) system was equipped with a stainless steel column packed with polyamine-bound silica (YMC gel PA-120; YMC, Kyoto, Japan). Each sample was injected into the column and eluted with a 0 to 100 mM gradient of sodium sulfate for 65 minutes at a flow rate of 0.5 ml/min. Then, 100 mM sodium tetraborate buffer (pH 9.0) containing 1% 2-cyanoacetamide was added to the column eluant at the same flow rate. The eluted sample was incubated at 145°C, and fluorescent products were detected by a fluoromonitor (excitation 346 nm, emission 410 nm). To calculate the amount of unsaturated disaccharides derived from products, authentic ones (Seikagaku Co.) were applied.

### Immunofluorescence Staining of U251-MG Cells

Expression of PTPζ on U251-MG cells was determined by immunofluorescence staining with anti-PTPζ antibody (H-300; Santa Cruz Biotechnology, Santa Cruz, CA) with fluorescein isothiocyanate-labeled goat F(ab′)2 fragment anti-rabbit IgG (H+L) (Beckman Coulter, Marseille, France). Nuclear staining was carried out by 4′,6-diamidino-2-phenylindole (DAPI). Control experiment was done by secondary antibody alone, and no specific staining was seen. Detection of GalNAc4S-6ST mRNA in U251-MG cells was carried out by *in situ* hybridization as described before.

### RNA Interference

Short-interfering RNAs (siRNA) targeting human GalNAc4S-6ST (siRNA ID no. 122847; 5′-GCAUCACAACUAGGAUUGAtt-3′) and Silencer Negative Control number 1 siRNA were purchased from Ambion (Austin, TX, USA), and the concentration was adjusted to 0.3 mg/ml with siRNA Suspension Buffer (Amaxa Biosystems, Koln, Germany). Before transfection, siRNAs were heated at 90°C for 1 minute, and then incubated at 37°C for 60 minutes. U251-MG cells were transiently transfected with siRNAs using an electropororator Nucleofector II device (Amaxa Biosystems), according to the manufacturer’s instructions. Briefly, 1.0×10^6^ of U251-MG cells plus 5 µl of 0.3 mg/ml GalNAc4S-6ST siRNA or the same volume of 50 µM control siRNA in 100 µl of Cell Line Nucleofector Kit V solution (Amaxa Biosystems) were transferred to an electroporation cuvette (Amaxa Biosystems) and electroporated following program T-020. Cells were then cultured with DMEM (Life Technologies) containing 10% FBS (HyClone Laboratories) in a humidified CO_2_ incubator at 37°C for 24 hours and subjected to an *in vitro* invasion assay. Suppression of GalNAc4S-6ST gene by the RNA interference was confirmed by quantitative RT-PCR as described above, and triplicate measurements were carried out.

### *In Vitro* Haptotaxic Migration Assay

An *in vitro* haptotaxic migration assay was carried out as described with minor modifications [23]. Briefly, the underside of 24-well Transwell polycarbonate membrane inserts with 3.0-µm pores (Corning, Corning, NY, USA) was coated with 12 µl of 5 mM Tris-HCl buffer containing 20 µg/ml PTN (R&D systems, Minneapolis, MN, USA), 20 µg/ml MK (R&D systems) or 20 µg/ml acidic FGF (aFGF) (R&D systems) for 2 hours. Inserts coated with 5 mM Tris-HCl served as controls. After adding 500 µl of DMEM containing 0.1% bovine serum albumin to the lower compartments, siRNA-transfected U251-MG cells were detached from culture dishes with enzyme-free Hanks’-based cell dissociation buffer (Invitrogen) and 2.0×10^4^ cells were transferred to the upper cell culture insert. Culture plates were then incubated for 5 hours. Cells failing to migrate through the membrane were removed by a cotton swab, and cells on the lower face of the insert, which had migrated through the membrane, were fixed with 4% buffered formalin followed by 1% crystal violet staining for 30 minutes. Migrating cells were counted in 5 randomly selected fields using a light microscope at x200 magnification. Assays were carried out in triplicate.

### Immunohistochemistry of Astrocytic Tumors

Expression of PTPζ, PTN, and MK in astrocytic tumor cells was examined by immunohistochemistry using anti-PTPζ antibody (clone 12; BD Biosciences, Franklin Lakes, NJ, USA), anti-human PTN antibody (R&D Systems), and anti-human MK antibody (R&D Systems), respectively. For secondary antibodies, HRP-conjugated goat anti-mouse IgG (Fab’) (MAX-PO(M)) (Nichirei, Tokyo, Japan) for PTPζ, and HRP-conjugated rabbit anti-goat immunoglobulins (Fab’) (MAX-PO(G)) (Nichirei) for PTN and MK were used. Counterstaining was carried out by hematoxylin. Control experiments were done by omitting primary antibodies, and no specific staining was seen. Tissue specimens containing more than 5% positively stained tumor cells were defined as positive, and others were classified as negative according to previously described criteria [24].

### Statistical Analysis

Statistical analysis was carried out using JMP 6 software (SAS, Cary, NC, USA) as described [25]. Significance with non-parametric distribution was evaluated by the Mann-Whitney *U* test for unpaired observations. Comparison of more than two groups was analyzed using the Kruskal-Wallis test. Analyses of 5-year patient survival based on GalNAc4S-6ST mRNA expression were carried out using the Kaplan-Meier method with the same software. Adjusted analysis of GalNAc4S-6ST mRNA expression and patient survival was carried out by multivariate data analysis using the Cox proportional hazards regression model. The stepwise procedure was adopted to select independent prognostic variables in multivariate analyses. The following variables were considered for statistical analysis: WHO histological grade, total resection or not, and postoperative Eastern Cooperative Oncology Group Performance Status (ECOG-PS). *P* values <0.05 were considered statistically significant.

## Results and Discussion

### GalNAc4S-6ST mRNA is Frequently Detected in Astrocytic Tumors

**Figure 1:**
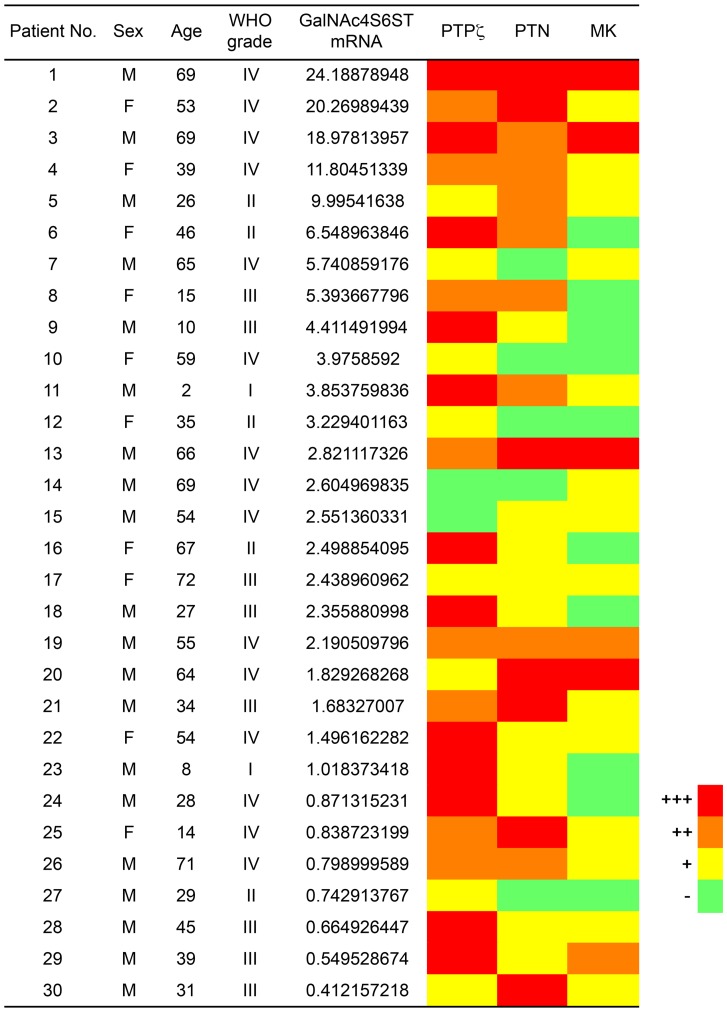
Expression of GalNAc4S-6ST mRNA, and proteins for PTPζ, PTN, and MK in astrocytic tumor patients. Patients are listed in descending order based on the fold expression of GalNAc4S-6ST mRNA calculated by setting the expression in human glioblastoma U251-MG cells to 1.0. Immunohistochemical expression of PTPζ, PTN, and MK in astrocytic tumor cells is indicated as a heat map, where negative <5%, weak +, moderate ++, strong +++.

Expression levels of GalNAc4S-6ST mRNA extracted from formalin-fixed, paraffin-embedded astrocytic tumor tissue sections were examined by quantitative RT-PCR using the comparative CT method. Defining the comparative CT value of GalNAc4S-6ST mRNA expression in human glioblastoma U251-MG cells as 1.0, the expression level of GalNAc4S-6ST transcripts in astrocytoma ranged from 0.412 to 24.189 (median (25–75 percentile) = 2.525 (0.982–5.480)). In particular, 23 patients showed stronger expression of GalNAc4S-6ST mRNA than U251-MG cells, while the expression of GalNAc4S-6ST mRNA in remaining 7 patients was weaker than U251-MG cells (Figure 1). Although the expression levels of GalNAc4S-6ST mRNA were variable among the patients, GalNAc4S-6ST mRNA was detected in all the patients examined. Since GalNAc4S-6ST is a key enzyme in CS-E biosynthesis, this result suggests that CS-E is produced in astrocytic tumors.

### CS-E is Synthesized in Astrocytic Tumors

To determine whether CS-E was produced in astrocytic tumors expressing GalNAc4S-6ST mRNA, levels of CS-E as well as other CS subunits in fresh tumor samples were quantitatively analyzed in 4 patients whose GalNAc4S-6ST mRNA expression level was determined. CS-E was detected in all patient samples, and levels ranged from 0.01 to 0.22 nmol/mg (Table 1). CS-E was most abundantly expressed in tissue from case No. 33, which showed strongest expression of GalNAc4S-6ST mRNA. It is noteworthy that CS-E was poorly formed in the tissue from case No. 34, in which the level of CS-A, precursor of CS-E, was also low. It seems reasonable, because GalNAc4S-6ST acts on CS-A as its specific substrate to produce CS-E [16], [17]. These results indicate that CS-E is indeed synthesized in human astrocytic tumors.

**Table 1:** Expression of chondroitin sulfates in astrocytoma tissues.

PatientNo.^1^	WHOgrade	GalNAc4S-6ST mRNA^2^	CS-E(nmol/mg)	CS-0S(nmol/mg)	CS-A(nmol/mg)	CS-C(nmol/mg)	CS-D(nmol/mg)
31	II	0.137	0.10	0.02	2.09	0.12	0.08
32	IV	0.049	0.12	0.48	1.39	0.23	0.01
33	II	0.621	0.22	0.21	5.48	0.27	0.19
34	II	0.109	0.01	n.d.^3^	0.21	0.03	0.02

1 Contiguous numbers continued from Figure 1.

2 Fold expression is calculated by setting the expression level seen in human glioblastoma U251-MG cells to 1.0.

3 not detected.

### GalNAc4S-6ST mRNA Serves as an Independent Biomarker of Poor Prognosis

**Figure 2:**
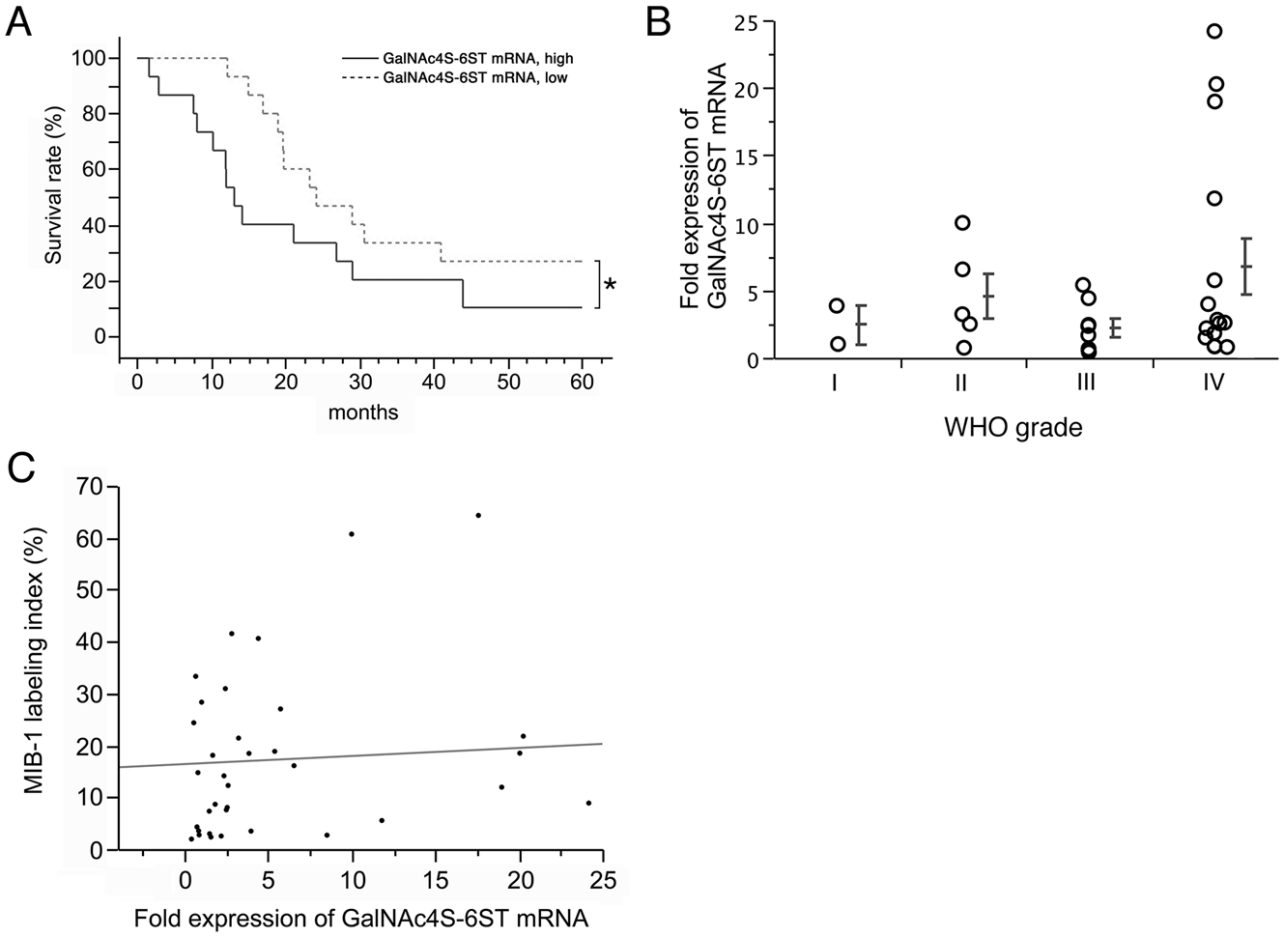
Association of GalNAc4S-6ST mRNA expressed in human astrocytic tumors with postoperative 5-year survival, WHO grades, and the MIB-1 labeling index. **A**) Expression level (fold expression) of GalNAc4S-6ST mRNA greater than the 2.5 cut-off is significantly associated with poor patient prognosis (*P = 0.0355; Kaplan-Meier curve). Expression of GalNAc4S-6ST mRNA in human glioblastoma U251-MG cells is defined as 1.0. **B**) Higher GalNAc4S-6ST expression was seen in tumors of higher WHO grade. However, no significant differences were seen among groups. Vertical bars indicate median and 25–75 percentiles. **C**) No significant relationship is seen in fold expression between GalNAc4S-6ST mRNA and the MIB-1 index.

Considering that CS-E is the preferred ligand of heparin-binding growth factors such as PTN and MK [3], [4], CS-E expressed by tumor cells may recruit PTN and MK, allowing cells to be more invasive and proliferative. To test the hypothesis that GalNAc4S-6ST expressed by astrocytoma could be associated with tumor progression, the relationship between GalNAc4S-6ST mRNA levels in astrocytic tumor and 5-year survival of the 30 tumor patients was statistically analyzed by the Kaplan-Meier method (Figure 2A). By defining the cut-off of tumor tissue expression of GalNAc4S-6ST as 2.5 employing a JMP 6 software program, we found that patients exhibiting high GalNAc4S-6ST expression in tumor had a significantly worse outcome than patients showing low expression of GalNAc4S-6ST (*P* = 0.0301).

We then carried out multivariate survival analysis using the Cox proportional hazards regression model comparing GalNAc4S-6ST mRNA expression with other clinicopathological prognostic factors, such as WHO tumor grade, total resection, and Eastern Cooperative Oncology Group Performance Status (ECOG-PS) after surgery versus patient survival (Table 2). Significantly, GalNAc4S-6ST mRNA was shown to be an independent prognostic factor for astrocytic tumor progression, since the adjusted hazard for GalNAc4S-6ST mRNA-high expression patients versus GalNAc4S-6ST mRNA-low expression patients was 15.64, with a 95% confidence interval (95% CI) of 4.27 to 71.91 (*P*<0.0001). The same analysis showed that WHO tumor grade was another independent indicator of poor prognosis (Table 2).

**Table 2:** Multivariate survival analysis for astrocytic tumors.

Variable	Hazard ratio	95% Confidence interval		*P* value
		Lower	Upper	
WHO grade				
> II vs = II	213.08	18.31	4312.00	<0.0001
Total resection	0.098	0.019	0.404	0.0008
ECOG-PS^1^ (after surgery)				
>1 vs ≤1	0.713	0.212	2.53	0.591
GalNAc4S-6ST mRNA expression^2^				
high vs low	15.64	4.27	71.91	<0.0001

1 ECOG-PS, Eastern Cooperative Oncology Group Performance Status.

2 Expression level (fold expression) of GalNAc4S-6ST mRNA greater than the 2.5 cut-off is defined as high, and other as low.

We next analyzed GalNAc4S-6ST mRNA expression relative to WHO tumor grade and the MIB-1 labeling index. Although a significant difference was not observed, expression of GalNAc4S-6ST mRNA in glioblastoma (WHO grade IV) was higher than that seen in anaplastic astrocytoma (WHO grade III) (*P* = 0.3705) (Figure 2B). On the other hand, expression levels of GalNAc4S-6ST mRNA were not correlated with tumor cell proliferation, as assessed by the MIB-1 index, indicating that GalNAc4S-6ST likely has no effect on proliferation of tumor cells (R^2^ = 0.0047) (Figure 2C).

### GalNAc4S-6ST is Transcribed in Astrocytic Tumor Cells

**Figure 3:**
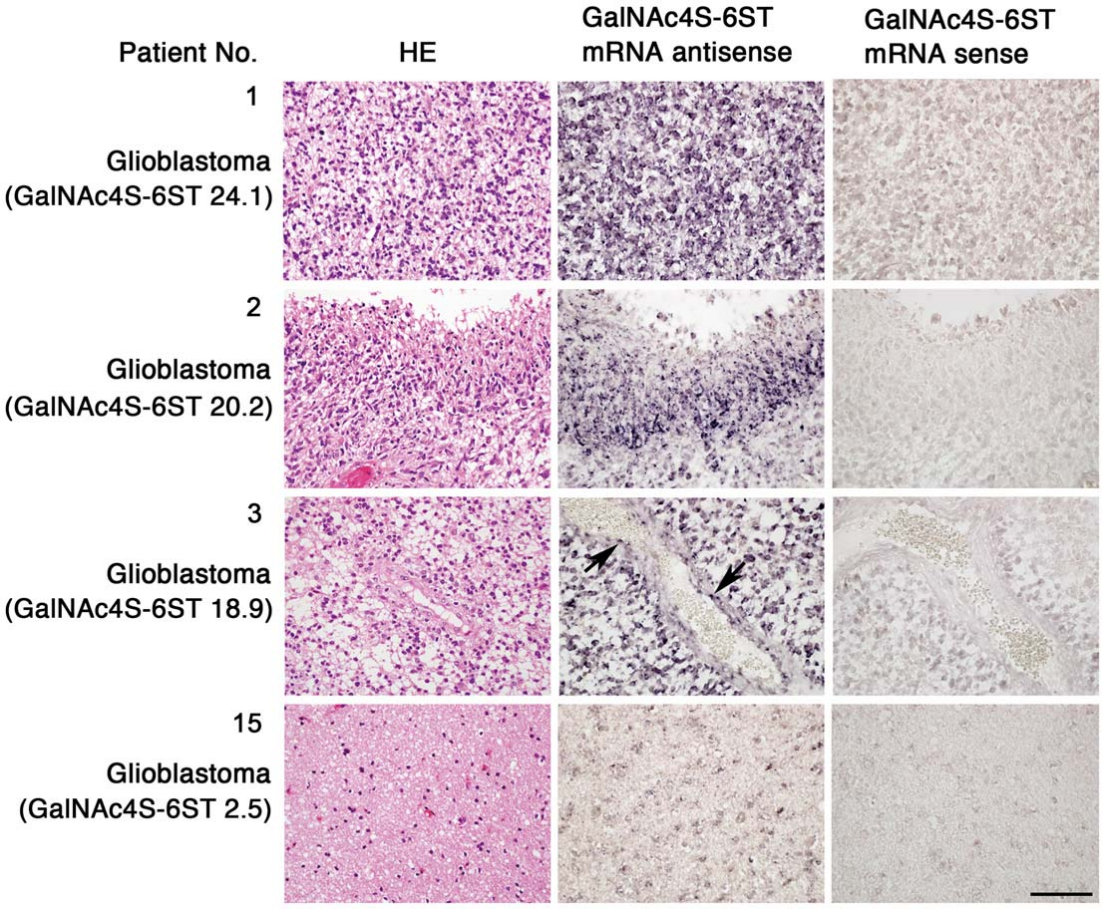
Expression of GalNAc4S-6ST mRNA in astrocytic tumor cells as revealed by *in situ* hybridization. GalNAc4S-6ST transcripts are detected in the cytoplasm of astrocytic tumor cells. Note that signal intensity for GalNAc4S-6ST mRNA is associated with expression level (fold expression) of GalNAc4S-6ST mRNA as determined by quantitative RT-PCR (shown in parentheses). Proliferating tumor endothelial cells also express GalNAc4S-6ST mRNA (shown by arrows). Sense probes serve as controls. Patient number in Figure 1 is indicated. Scale bar = 100 µm.

To determine which cells express GalNAc4S-6ST mRNA in astrocytic tumors, *in situ* hybridization was performed using a DIG-labeled GalNAc4S-6ST RNA probe on tissue specimens from 4 patients showing different GalNAc4S-6ST mRNA expression levels, as determined by quantitative RT-PCR. Positive signals for GalNAc4S-6ST mRNA were detected in astrocytic tumor cell bodies in all patients examined (Figure 3). Notably, signal intensity for GalNAc4S-6ST mRNA mirrored expression levels of GalNAc4S-6ST mRNA determined by real-time RT-PCR, indicating that signal intensity obtained by *in situ* hybridization is quantitative as shown previously [21]. Previously, Kato et al (2008) demonstrated that the transcripts of sulfotransferases including KSGal6ST, GlcNAc6ST-1, and GlcNAc6ST-5, that are responsible for sulfation of kertan sulfate (KS), are significantly up-regulated in glioblastoma compared with diffuse astrocytoma and anaplastic astrocytoma isolated from brain tumor patients using quantitative PCR analysis, suggesting the association of KS with progression of astrocytic tumors [26]. However, detection of GalNAc4S-6ST mRNA involved in sulfation of CS-E in glioblastoma cells by using *in situ* hybridization had not been reported. In addition to tumor cells, the present study revealed that GalNAc4S-6ST transcripts were also detected in proliferating endothelial cells in tumors (Figure 3). Recently, it has been demonstrated that biglycan, a small leucin-rich repeat proteoglycan, is specifically expressed in tumor endothelial cells [27]. It is of great significance to determine whether biglycan exhibiting CS-E is expressed on the tumor endothelial cells.

### Haptotaxic Migration of U251-MG Cells Mediated by PTN and MK is Decreased by GalNAc4S-6ST Knockdown

**Figure 4:**
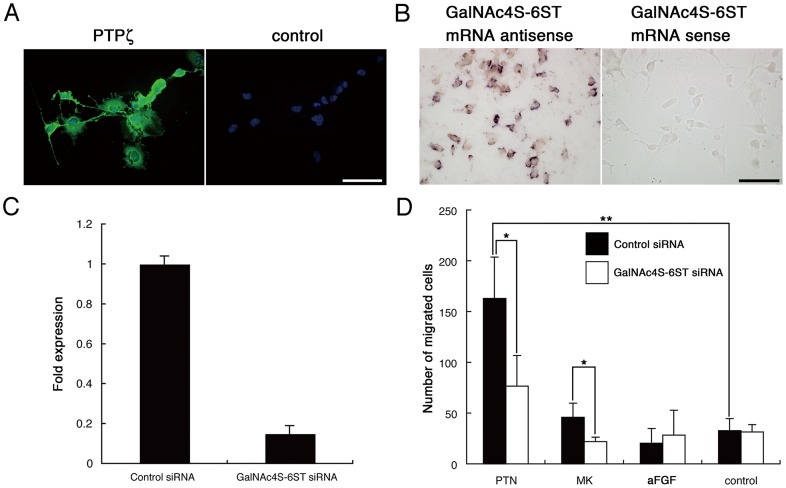
Effects of GalNAc4S-6ST siRNA on human glioblstoma U251-MG cells. A ) U251-MG cells express PTPζ. Control staining with secondary antibody alone is also shown. DAPI for nuclear staining. Scale bar = 100 µm. **B**) GalNAc4S-6ST mRNA is detected in U251-MG cells using the antisense probe. The sense probe reveals no specific staining. Scale bar = 100 µm. **C**) Expression levels of GalNAc4S-6ST mRNA are significantly reduced in U251-MG cells transfected with GalNAc4S-6ST siRNA compared with control siRNA (*P*<0.01). Data represent the mean ± SD from triplicate measurements. **D**) Haptotaxic migration of U251-MG cells transfected with control siRNA is significantly enhanced in the presence of PTN compared with the absence of PTN (**P<0.001). Enhancing effects on haptotaxic migration of U251-MG cells by PTN and MK are significantly suppressed when U251-MG cells are transfected by GalNAc4S-6ST siRNA (*P<0.05). aFGF treatment has no effect on haptotaxic migration of U251-MG cells transfected by control siRNA or GalNAc4S-6ST siRNA. Data represent the mean ± SD from triplicate analyses.

DNA microarray analysis by Müller et al (2003) revealed that the brain-specific chondroitin sulfate proteoglycan protein tyrosine phosphatase ζPTPζ) and its ligand PTN are upregulated in glioma compared with normal brain [28]. Binding of PTN and the related molecule MK to PTPζis reportedly mediated by CS-E attached to PTPζ [29]. As shown previously by Ulbricht et al (2006) [23] and confirmed here, human glioblastoma U251-MG cells express PTPζ(Figure 4A). We also revealed that U251-MG cells expressed GalNAc4S-6ST mRNA using *in situ* hybridization (Figure 4B). Thus, we employed a modified Boyden chamber method to determine whether CS-E enhances haptotaxic migration of glioblastoma U251-MG cells in presence of PTN or MK. Haptotaxic stimulation of migration by PTN was most effective in control siRNA-transfected U251-MG cells, while that enhancing effect was cancelled when GalNAc4S-6ST siRNA, which reduced GalNAc4S-6ST mRNA to 20% of control levels (Figure 4C), was introduced instead of control siRNA (Figure 4D). Although the enhancing effect was not so robust as with PTN, similar results were obtained using MK (Figure 4D). However, aFGF, that is hardly bound to CS-E [3], had no effect on haptotaxic stimulation of migration (Figure 4D). These results suggest that PTN and MK enhance U251-MG cell migration by binding to CS-E. The PTPζ phosphatase is expressed on the cell surface as a monomer, and its phosphatase domain is active in the absence of PTN or MK [29]. However, upon binding to PTN or MK ligands, PTPζ dimerizes, leading to inactivation of the phosphatase domain and a net increase in intracellular tyrosine phosphorylation. Previously, Ulbricht et al. demonstrated that reduced haptotactic migration of U251-MG cells induced by PTN was achieved by siRNA-mediated knock down of PTPζ [23]. Although it was not shown whether CS-E directly attaches to PTPζ expressed on U251-MG cells, these results suggest that CS-E regulates PTPζ phosphatase activity on astrocytic tumor cells in the presence of PTN and MK.

### PTPζ and its Ligands PTN and MK are Frequently Expressed in Astrocytic Tumor Cells

**Figure 5:**
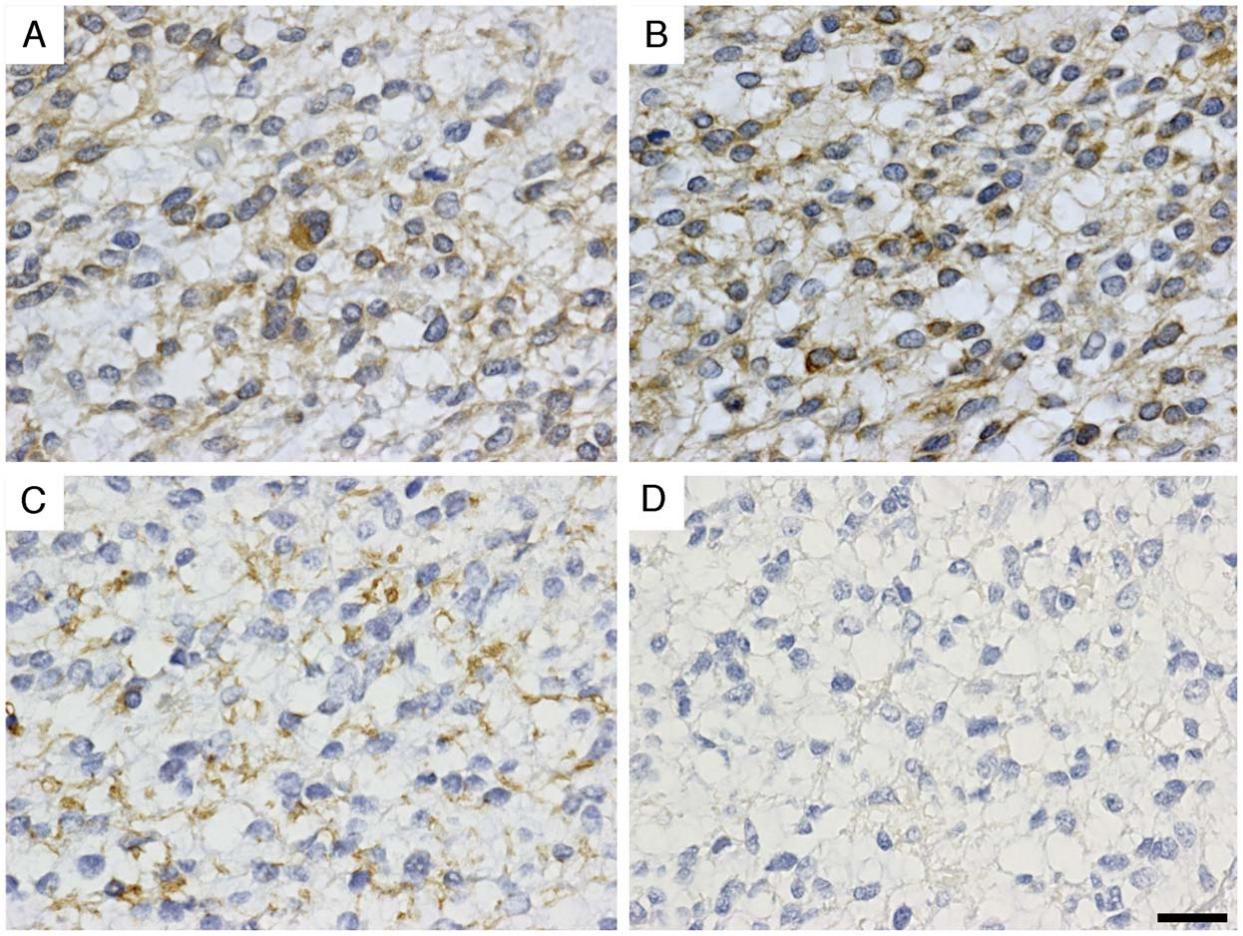
Expression of PTPζ, PTN, and MK in human glioblastoma. Immunohistochemistry using antibodies specific for PTPζ (**A**), PTN (**B**), and MK (**C**) are shown. Control indicates secondary antibody only (**D**). Patient No. 1 in Figure 1 is shown. Scale bar = 20 µm.

Lastly, we examined whether PTPζ and its ligands, PTN and MK, are expressed in human astrocytic tumor cells. Immunohistochemistry with specific antibodies revealed that PTPζ is expressed in the cell body and cytoplasmic processes of tumor cells in 28 (93.3%) of 30 patients (Figures 1 and 5A). PTN and MK were expressed in tumor cells of 25 (83.3%) and 20 (67.6%) of 30 patients, respectively (Figure 1). Notably, PTN was predominantly expressed in the cell body of tumor cells, while MK was largely expressed in cytoplasmic processes (Figure 5B and C).

In summary, these results indicate that CS-E, which is formed by GalNAc4S-6ST on astrocytic tumor cells, allows tumor cells to migrate more robustly upon binding to PTN and/or MK secreted from astrocytic tumor cells in a paracrine and/or autocrine manner, potentially through inactivation of the PTPζ phosphatase domain. Since higher expression of GalNAc4S-6ST mRNA is associated with poor patient prognosis, our results provide a molecular rationale for development of new therapy for astrocytic tumors targeting GalNAc4S-6ST.
